# Unveiling the immunopharmacological mechanisms of Danggui Yinzi (DGYZ) in treating chronic urticaria: insights from network pharmacology and experimental validation

**DOI:** 10.1186/s13020-025-01137-7

**Published:** 2025-06-09

**Authors:** Xu-rui Wang, An-jing Chen, Chang-cheng Hou, Yue-yue Wang, Jing Guo, Ming-yue Li

**Affiliations:** 1 Department of Chinese Medicine Surgery, Sichuan Provincial People’s Hospital, University of Electronic Science and Technology of China, Chengdu, China; 2 Chengdu University of Traditional Chinese Medicine, Chengdu, China; 3 Jiangsu Province Hospital of Traditional Chinese Medicine Chongqing Hospital, Chongqing, China; 4 Dermatological Department, Hospital of Chengdu University of Traditional Chinese Medicine, No.39, 12 Qiao Road, Jinniu District, Chengdu, Sichuan China; 5 Special Needs Outpatient Department, Hospital of Chengdu University of Traditional Chinese Medicine, No.39, 12 Qiao Road, Jinniu District, Chengdu, Sichuan China

**Keywords:** Danggui Yinzi, Chronic urticaria, Network pharmacology, Immune mechanism

## Abstract

**Background:**

Chronic urticaria (CU), a prevalent and often debilitating allergic skin disorder, is primarily triggered by mast cell degranulation. Danggui Yinzi (DGYZ), a traditional Chinese medicine formula, has been employed to treat pruritic conditions. However, the molecular mechanisms underlying its effects in CU remain unclear. This study aimed to investigate the immunopharmacological mechanisms of DGYZ in CU, hypothesizing that it modulates immune responses through its bioactive components, which is critical for the development of novel therapeutic agents.

**Methods:**

Ultra-performance liquid chromatography coupled with quadrupole time-of-flight tandem mass spectrometry (UPLC-Q-TOF–MS) was used to identify the active compounds in DGYZ. In vivo, BALB/c mouse models of DNP-IgE/DNFB-induced CU were established and grouped into Normal Control (NC), Model, various-dose DGYZ, and Loratadine groups. Post-treatment, immunopharmacological parameters were assessed, and skin tissue was collected for histopathological analysis, mast cell quantification, and immunohistochemistry to evaluate the impact on immune cells and molecules. Serum levels of inflammatory cytokines (TNF-α, IL-6) were quantified using ELISA kits. In vitro, the human mast cell line LAD2 was pretreated with key active components of DGYZ (Quercetin and Paeoniflorin) at different concentrations before mast cell degranulation was induced. Degranulation markers (β-HEX, HIS) and the expression of proteins in immune-related signaling pathways (PI3K-Akt, TLR4) were then measured.

**Results:**

A total of 38 active components were identified in DGYZ. In vivo, DGYZ inhibited mast cell degranulation, blue spot reactions, and skin damage in mice. It also decreased the levels of inflammatory cytokines (TNF-α, IL-6) and suppressed the activation of associated signaling pathways. In vitro, both Quercetin and Paeoniflorin reduced mast cell degranulation and the activation of TLR4 and PI3K-Akt pathways.

**Conclusion:**

This study, employing UPLC-Q-TOF–MS and both in vivo and in vitro experiments, provides a comprehensive analysis of the mechanism of DGYZ in CU. The findings indicate that DGYZ exerts therapeutic effects in CU by modulating immune responses. This research lays the foundation for a deeper understanding of its immunopharmacological mechanisms, potentially aiding the development of novel drugs and therapeutic strategies for CU management.

**Graphical Abstract:**

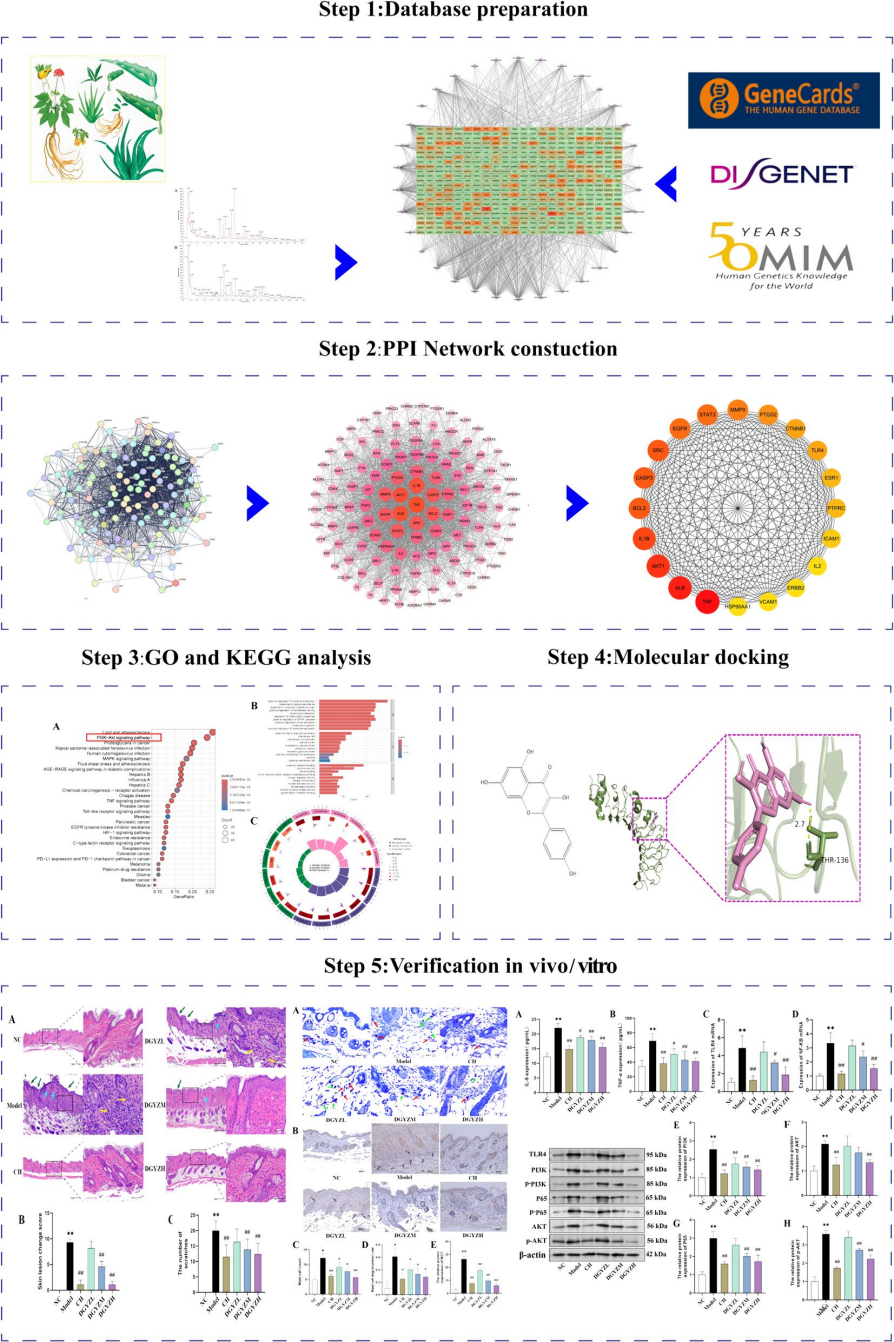

## Introduction

Urticaria, one of the most prevalent allergic skin disorders, is characterized by transient increases in vascular permeability within the mucocutaneous skin, triggered by various allergic stimuli. This results in a localized edematous response, leading to erythema and wheals [1, 2]. Chronic urticaria (CU) is defined by recurrent episodes lasting more than six weeks, with symptoms occurring at least twice per week [3]. CU is divided into two subtypes: chronic inducible urticaria (physical urticaria, CIU) and chronic idiopathic urticaria (chronic spontaneous urticaria, CSU) [4]. The pathogenesis of urticaria is closely related to the immune system. The core link is the degranulation of mast cells, which involves multiple immune-related factors. Antigens are processed by antigen-presenting cells, which prompt Th2 cells to stimulate B cells to produce antigen-specific IgE. IgE binds to receptors on the surface of mast cells, triggering degranulation and the release of inflammatory mediators, which are involved in the pathogenesis of chronic spontaneous urticaria and various inducible urticarias. Multiple inflammatory mediators are involved in the immunopathogenesis of urticaria. Chemokines [5] cytokines [6], substance P [7], prostaglandin E2 [8], etc., play roles in the pathogenesis of urticaria by inducing, stimulating, or regulating mast cell degranulation respectively, and are involved in the immune process of the disease.

Although antihistamines such as loratadine are first-line treatment drugs, the symptom control effect is still unsatisfactory for some patients even when they use the conventional dose or an increased dose [4, 9]. This is because antihistamines only target histamine-mediated inflammation and have limited effects on the complex mechanisms of the disease. Moreover, antihistamines have a variety of adverse reactions, such as drowsiness, fatigue, headache, dry mouth, and gastrointestinal discomfort. In severe cases, liver function abnormalities and arrhythmias may occur [10, 11]. There are also restrictions on the use of antihistamines in special populations. There is insufficient safety data for pregnant and lactating women. Elderly patients have a higher risk of adverse reactions when using antihistamines due to reduced liver and kidney function [12].

Dang-Gui-Yin-Zi (DGYZ), a traditional Chinese medicine (TCM) first recorded in"Yan Shi Ji Sheng Fang"during the Southern Song Dynasty, Which has a long history of clinical use to treat pruritus, neurodermatitis, psoriasis, and urticaria [13]. According to TCM principles, pruritus results from deficiencies in qi and blood, leading to insufficient nourishment of the skin and the manifestation of wind-induced skin pruritus. DGYZ is believed to replenish qi and blood, dispel wind, and alleviate itching, thus making it a commonly used remedy for urticaria [14]. Recent investigations into chronic urticaria (CU) have demonstrated that Danggui Yinzi (DGYZ) exerts therapeutic potential surpassing traditional antihistamines through the synergistic effects of its multi-component, multi-target profile. In vivo and in vitro studies confirm that DGYZ inhibits excessive mast cell activation via mechanisms including enhancing cellular autophagy—evidenced by upregulated LC3B and p62 expression at both mRNA and protein levels [15]—and modulating inflammatory cascades. This results in reduced release of key mediators such as IL-33, histamine, and TNF-α, while restoring Th1/Th2 immune balance to suppress aberrant type I hypersensitivity in CU [16]. Contrasting with single-target antihistamines (e.g., loratadine), which solely address histamine-mediated pathways, DGYZ offers systemic intervention across the complex pathogenesis of CU. Its mechanisms encompass: (1) attenuation of mast cell degranulation by downregulating IgE receptor expression [14], 2 inhibition of innate immune hyperactivation via the TLR4/MyD88 signaling axis [17], and (3) correction of Th2-polarized adaptive immune dysregulation—thereby interrupting the perpetuation of chronic inflammation [18]. Preclinical data further highlight DGYZ’s safety profile, as it elicits no significant sedation, arrhythmia, or other adverse effects commonly associated with antihistamines [19]. The multi-component synergy of DGYZ also mitigates toxicity risks inherent to long-term monotherapy with isolated compounds. These findings transcend the limitations of single-target therapies, elucidating an innovative paradigm of"multi-pathway immune homeostasis regulation"by DGYZ in CU management and paving the way for developing safe, efficacious therapeutic strategies for this disorder. However, the precise mechanisms underlying the itch-relieving effects of DGYZ remain to be fully elucidated.

Network pharmacology (NP), a concept introduced by Hopkins AL in [20], has emerged as a powerful tool to explore the complex mechanisms induced by natural drugs [20, 21]. NP allows for the visualization of complex interaction networks, elucidating relationships between drugs, targets, and diseases, and revealing synergistic effects of multi-component treatments. Consequently, NP is widely used to investigate the"drug-target-disease"interaction network [22]. Although there have been some studies on the treatment of pruritic skin diseases with Danggui Yinzi (DGYZ) using network pharmacology methods [23, 24], compared with previous studies, our research has many new contributions. We have revealed that DGYZ can regulate the immune response in CU. Specifically, it inhibits mast cell degranulation and reduces the levels of inflammatory cytokines such as TNF-α and IL-6, which was not emphasized in previous studies. Thirty-eight active components in DGYZ were identified by UPLC-Q-TOF–MS, with quercetin and paeoniflorin being key components. It was also found that these components act on proteins in the PI3 K-Akt and TLR4 signaling pathways, such as PIK3 C2G, AKT1, and TLR4. However, these specific targets and their relationships were not fully explored in previous studies. The predicted targets were subsequently validated through in vivo and in vitro experiments. Our findings provide novel insights into therapeutic strategies and the underlying signaling mechanisms for managing CU.

## Methodologies and materials

### Identification of DGYZ drug ingredients (UPLC-Q-TOF–MS/MS analysis)

HPLC-Q-TOF–MS was utilized for a comprehensive analysis of the drug constituents in DGYZ. DGYZ is composed of several herbal ingredients, including *Angelica archangelica* L. (Dang-Gui), *Kochia scoparia* (L.) Schrad. (Di-Fu-Zi), *Ligusticum sinense* Olive. (Chuan-Xiong), *Cynanchum otophyllum* C.K. Schneid. (Bai-Shao), *Saposhnikovia divaricata* (Turcz.) Schischk. (Fang-Feng), *Astragalus membranaceus* Fisch. ex Bunge (Huang-Qi), *Spatholobus suberectus* Dunn (Ji-Xue-Teng), *Glycyrrhiza uralensis* Fisch. ex DC. (Gan-Cao), *Taxillus chinensis* (DC.) Danser (Sang-Ji-sheng), *Rehmannia glutinosa* (Gaertn.) DC. (Di-Huang), and *Pleuropterus multiflorus* Turcz. ex Nakai (He-Shou-Wu). All plant names were verified against the World Flora Online database (2024/11/24) to ensure consistency with the latest taxonomic revisions. Approximately 1 g of DGYZ was ground through a 4-mesh sieve, accurately weighed, and placed in a sealed tapered bottle. To this, 50 mL of 70% methanol was added, and the mixture was weighed again before undergoing ultrasonic extraction for 30 min. The solution was removed, cooled, and reweighed to compensate for any weight loss by adding 70% methanol. The solution was mixed thoroughly and filtered through a 0.22 µm membrane, and the resultant filtrate was collected. Chromatographic separation was performed using a Thermo Scientific AccucoreTM C18 column (3 mm × 100 mm, 2.6 μm). The mobile phase consisted of 0.1% formic acid in water (A) and 0.1% formic acid in acetonitrile (B), with a flow rate of 0.5 mL/min and a column temperature set at 30 °C. A 2 µL sample volume was injected, with the following gradient elution: 0–10 min, B: 5–10%; 10–20 min, B: 10–25%; 20–35 min, B: 25–50%. Electrospray ionization (ESI) was applied in positive ion mode. The operational parameters were optimized as follows: spray voltage at 3.2 kV, ion source temperature at 350 °C, sheath gas flow rate at 35 arb, auxiliary gas flow rate at 10 arb, and ion transport tube temperature at 320 °C. Raw data were processed using Compound Discoverer 3.0 software, with unknown compounds identified through its wizard and method templates. Compound analysis involved comparing the filtered ions with a comprehensive database of compounds, reference standards, and pertinent literature.

### Analysis based on NP

#### Active ingredient and disease target prediction

Compound structure files were sourced from the PubChem database (https://pubchem.ncbi.nlm.nih.gov/) and imported into the SwissTarget Prediction database (http://www.swisstargetprediction.ch/) to predict potential targets. Homo sapiens was selected for target prediction with a filtering criterion of Probability > 0. Target data were retrieved from the following databases: The Human Gene Database (GeneCards: https://www.genecards.org), a comprehensive resource of genomic, proteomic, and functional data on human genes [25], Online Mendelian Inheritance in Man (OMIM: http://omim.org/), a curated resource for human genes and genetic disorders [26], and Disease Gene Network (DisGeNET: https://www.disgenet.org/), a platform containing one of the largest collections of genetic elements and variations linked to human diseases [27]. Gene data related to CU were extracted using the search term “chronic urticaria.” After consolidating targets from these repositories, redundancies were removed, and shared targets between DGYZ and CU were identified using Venn Diagrams (http://bioinformatics.psb.ugent.be/webtools/Venn/) to explore overlapping targets between the drug and the disease [28].

#### Protein–protein interaction (PPI) network development

The intersecting targets were imported into STRING (http://stitch.embl.de/) for PPI analysis. STRING facilitates the retrieval of known PPI associations, providing insights into complex regulatory networks in biological systems [29]. The analysis was configured for"multiple proteins,"with the organism set to"*Homo sapiens*"and a minimum interaction score threshold of ≥ 0.4, while other settings were kept at their default values. The resulting PPI networks were visualized using Cytoscape.

#### GO and KEGG pathway enrichment analyses

Gene Ontology (GO) and Kyoto Encyclopedia of Genes and Genomes (KEGG) pathway enrichment analyses were conducted using the ClusterProfiler package (version 3.5.1) in R software (version 4.1.0) [30]. GO terms and KEGG pathways with a q-value ≤ 0.05 were considered statistically significant. The top 10 GO terms from each category—cellular component (CC), biological process (BP), and molecular function (MF)—as well as the top 20 KEGG pathways, were selected for further analysis. The enriched targets within the major signaling pathways were visualized using Cytoscape (version 3.10.1) based on the results of these analyses.

#### Molecular docking (MD)

The 2D structures of small molecular ligands were retrieved from the PubChem database and converted into 3D structures using ChemOffice 20.0 software, saving them as mol2 files. High-resolution crystal structures of the protein targets were obtained from the RCSB PDB database (http://www.rcsb.org/) to serve as receptors for MD simulations. Water molecules and phosphate groups were removed from the protein structures using PyMOL 2.6.0 software, and the cleaned structures were saved as PDB files. Energy minimization of the compounds was performed using Chem3D software, which also preprocessed the target proteins and identified potential active binding pockets. MD simulations were carried out using Autodock Vina, performing 10 separate runs to optimize the binding energy. Binding interactions were assessed based on the optimal binding energy, with results visualized using PyMOL.

### Experimental validation

#### Reagents

The Angelica Decoction used in the study was provided by China Resources Sanjiu Pharmaceutical Co., Ltd. Anti-2,4-dinitrophenyl-specific IgE (DNP-IgE) was supplied by Sigma-Aldrich (St. Louis, MO, USA), while DNFB was sourced from Macklin (Shanghai, China). Loratadine tablets (Keratane, 10 mg × 12 tablets), manufactured by Bayer Pharma (Shanghai) Co., Ltd. (SINopol H10970410), were also utilized.

#### Preparation of DGYZ extract

The 12 herbs used in this study were obtained from the Pharmacy Department of the Hospital of Chengdu University of Traditional Chinese Medicine (Chengdu, China). Specimen certificates for these herbs are archived at the Affiliated Hospital of Chengdu University of Traditional Chinese Medicine. The herbs included Dang-Gui, Di-Fu-Zi, Chuan-Xiong, Bai-Shao, Fang-Feng, Huang-Qi, Ji-Xue-Teng, Gan-Cao, Can-Tui, Bai-Ji-Li, Di-Huang, and He-Shou-Wu, which were mixed in the ratio of 10:15:10:15:30:30:20:9:10:15:20:15(g). These herbs were mixed and boiled twice with purified water, and the water-based decoction was concentrated to 2 g/mL.

#### Animals

Sixty SPF-grade BALB/c mice (4–6 weeks of age, weighing 20 g ± 2 g) were obtained from Sichuan Lilesino Biotechnology Co., Ltd. (License No.: SCXK(Sichuan)2020–0030). The mice were housed in the Lilai Experimental Animal Center under controlled conditions (room temperature: 23 ± 1 °C, relative humidity: 60–65%) and provided with commercial pellet feed and drinking water ad libitum. After a 1-week acclimatization period, the mice were used for experimentation. This study was approved by the Committee on the Ethics of Animal Experiments of Sichuan Provincial People's Hospital (approval number: 2024–282) and adhered to the Guide for the Care and Use of Laboratory Animals issued by the National Institutes of Health (NIH). Efforts were made to minimize pain and suffering, with all experimental procedures following the animal care guidelines of Europe and respective home countries.

#### Animal model and treatments

After acclimatization, the 60 BALB/c mice (10 per group) were randomly assigned into six experimental groups: the Normal Control (NC) Group, which received a tail intravenous injection of normal saline (0.25 mL/mouse); the Model Group, which received a tail intravenous injection of monoclonal antibody (0.25 mL/mouse); the High-Dose DGYZ Group (DGYZH), which received monoclonal antibodies and 5.76 g/kg DGYZ daily; the Middle-Dose DGYZ Group (DGYZM), which received monoclonal antibodies and 2.88 g/kg DGYZ daily; the Low-Dose DGYZ Group(DGYZL), which received monoclonal antibodies and 1.44 g/kg DGYZ daily; and the Loratadine Group (CH), which received monoclonal antibodies and loratadine (0.9 mg/kg orally).DGYZ doses (high: 5.76 g/kg, middle: 2.88 g/kg, low: 1.44 g/kg) were determined based on clinical-to-animal dose conversion and preliminary validation. The high dose was calculated as 10 times the human equivalent dose via body surface area scaling [31]. Middle and low doses were set at 50% and 25% of the high dose to assess dose dependence. Acute toxicity tests confirmed safety (LD50 > 20 g/kg).To establish the urticaria lesion model, all mice, except the NC group, received a monoclonal antibody injection (0.25 mL/mouse via tail vein) on day 7. 24 h later, DNFB was applied to the back of the ear and the backs of the other groups were stimulated. From days 10 to 16, the DGYZ and CH groups were treated orally with loratadine or DGYZ solution (as per their group doses) for seven consecutive days. On day 16, 50 µL of the drug was applied again, and the number of scratches within 20 min was recorded. On day 17, all mice were humanely euthanized with pentobarbital sodium (60 mg/kg, i.p.). Blood (500 µL) was drawn from the eyeball, and skin tissue from the shaved back area was collected for analysis.

#### Cell experiments

For the in vitro studies, the human mast cell line LAD2 (FenghBio, Changsha, China) was cultured in 1640 medium (Procell, Wuhan, China). Cells were digested with trypsin and passaged when they reached 70–80% confluence. The cell groupings were as follows: (i) Control + Que/Kae (12.5, 25, 50, 100 µM), Model + Que/Kae (12.5, 25, 50, 100 µM); (ii) Control, Model, Que/Kae -L (25 µg/mL), Que/Kae -H (50 µg/mL). Que/Kae was pretreated for 3 h at the specified concentration gradient, and mast cell degranulation was induced by adding 100 ng/mL DNP-BSA (EMD Chemicals, Gibbstown, NJ) and incubating for 24 h at 37 °C and 5% CO2, as described by Zhang et al. [32].

#### Histopathological analysis

For pathological analysis, skin tissues from the shaved area on the backs of the mice were fixed in 10% paraformaldehyde (Chengdu Cologne Chemical Co., Ltd., Chengdu, China) for 24 h at 4 °C. After fixation, the tissues were processed and embedded in paraffin (Shanghai Hualing Rehabilitation Equipment Factory, Shanghai, China). Sections (5 µm thick) were stained with hematoxylin (Wuhan Seville Biotechnology Co., Ltd., Wuhan, China; cat. no. C191204) for 5 min at room temperature and eosin (H&E; Zhuhai Bezo Biotechnology Co., Ltd., Zhuhai, China) for 2 min to evaluate histopathological changes. The slides were observed under light microscopy (IX71; Olympus Corporation) at × 100 magnification, using a × 10 eyepiece and × 10 objective, with three fields per slide examined, and images were captured by a pathologist.

#### Mast cell detection

To assess mast cell density and degranulation, skin specimens were fixed overnight in 10% paraformaldehyde (Chengdu Cologne Chemical Co., Ltd., Chengdu, China), followed by dehydration in ethanol, paraffin embedding, and sectioning into 5 µm-thick slices. These sections were stained with 1% toluidine blue (TB) solution (Shanghai Ruji Biotechnology Development Co., Ltd., Shanghai, China; Lot No.: 190410) and preheated to 50 °C for 20 min. After rinsing with distilled water, sections were briefly exposed to 0.5% glacial acetic acid for 5 s, dehydrated through an ethanol gradient, cleared with xylene, and mounted with neutral gum. Mast cells were counted under a microscope (OLYMPUS, Tokyo, Japan) by randomly selecting three fields per section at 100 × and 400 × magnification.

#### Determination of serum TNF-α, IL-6 levels

Blood samples from the UL mice were centrifuged at 3000 × g to collect serum, which was stored at − 80 °C until further analysis. Serum concentrations of IgE, TNF-α, IL-6, and HIS were quantified using respective ELISA kits (Shanghai Zhuocai Biotechnology Co., Ltd., Shanghai, China), with optical density (OD) measured at 450 nm on a microplate reader (Meigu Molecular Instrument Co., Ltd., Shanghai, China; SpectraMAX Plus384). β-HEX and HIS levels were determined based on their standard curves.

#### Immunohistochemistry (IHC)

Fixed tissue was processed using an automated dehydrator, embedded, and sectioned before being placed in a staining cylinder. Endogenous peroxidase activity was blocked with 3% methanol hydrogen peroxide for 10 min, followed by three PBS washes. Sections underwent microwave antigen retrieval in citrate buffer (pH 6.0) at 95 °C for 10 min. Blocking was performed with bovine serum albumin (BSA) at room temperature for 20 min (cat. No. GC305010; Wuhan Xavier Biotechnology Co., Ltd.). MCT primary antibody (dilution 1:100; cat. No. AH339; Shanghai Beyotime Biotechnology Co., Ltd.) was applied overnight at 4 °C. The secondary antibody (HRP-conjugated goat anti-rabbit, cat. No. GB22303; Wuhan Xavier Biotechnology Co., Ltd.) was incubated at 37 °C for 30 min. Following PBS washes, the DAB color development kit (cat. No. ZL1-9018; Beijing Zhongshan Jinqiao Biology Co., Ltd.) was used at room temperature for 2 min, followed by distilled water rinsing, hematoxylin counterstaining for 20 s, dehydration, and mounting with neutral gum. Specimens were examined under optical microscopy, with three randomly selected fields recorded. Semi-quantitative analysis of MCT levels was performed using Image Pro Plus 6.0 software (Media Cybernetics, Inc.) to calculate the integrated optical density average at 400 × magnification.

#### Western blot (WB) analysis

Skin tissue from mice was dissected into 30 mg pieces using sterile, disinfected scissors and lysed in 300 µL of radioimmunoassay buffer containing protease and phosphatase inhibitors. The tissue was disrupted via ultrasound, incubated on ice for 10 min, and the protein lysate was collected. The lysate was centrifuged at 16,000 × g at 4 °C for 30 min. Protein extraction was performed using RIPA buffer, and protein concentration was quantified by the BCA assay. SDS-PAGE was used to separate 20 µg of total protein per lane, followed by transfer onto PVDF membranes. Membranes were blocked with 5% non-fat milk for 1 h and then incubated overnight at 4 °C with primary antibodies: Anti-AKT (1:2000; Rabbit; No. A18675; abclonal), Anti-phosphorylation-AKT (p-AKT, 1:1000; Rabbit; No. AP0637; abclonal), Anti-PI3 K (1:1000; Rabbit; No. AP0427; abclonal), Anti-phosphorylation-PI3 K (p-PI3 K, 1:1000; Rabbit; No. AP0427; abclonal), Anti-p65 (1:1000; Rabbit; No. A19653; abclonal), Anti-phosphorylation-p65 (p-p65, 1:1000; Rabbit; No. AP1460; abclonal), Anti-TLR4 (1:1000; Rabbit; No. ab218987; abcam), and β-actin (1:100000; Rabbit; No. AC026; abclonal). After incubation, membranes were washed with TBST and incubated with a secondary antibody (Goat Anti-Rabbit IgG (H + L) HRP, 1:5000; cat. No. S0001; Affbiotech) at 37 °C for 1 h. Immune complexes were visualized using Pierce™ ECL Western Blotting Substrate (Thermo Fisher).

#### Real-time polymerase chain reaction analysis (RT-PCR)

RNA was extracted from tissue samples using the Total Tissue RNA Extraction Kit (YEASEN, 19221ES50), and cDNA was synthesized using the PrimeScript Reverse Transcription Kit (Takara, RR047 A) following the manufacturer's instructions. Quantitative PCR was conducted using the SYBR Premix Ex Taq^™^ Kit (Takara, RR820 A), with all RT-PCR experiments performed in triplicate. Gene sequences were sourced from the National Center for Biotechnology Information (NCBI) database, and gene-specific primers were designed and validated using Primer Premier. Custom primers were synthesized by Shanghai Shenggong Bioengineering Technology Service Co., Ltd., and purified via ULTRAPAGE.

#### Statistical analysis

Statistical analysis was performed using GraphPad software. t-tests were applied to compare mean values (± standard error of the mean, SEM) from at least triplicate samples across two independent experiments between co-cultured and reference control melanoma cells. A p-value of less than 0.05 (p < 0.05) was considered statistically significant.

## Results

### Main active ingredients of DGYZ

**Fig. 1:**
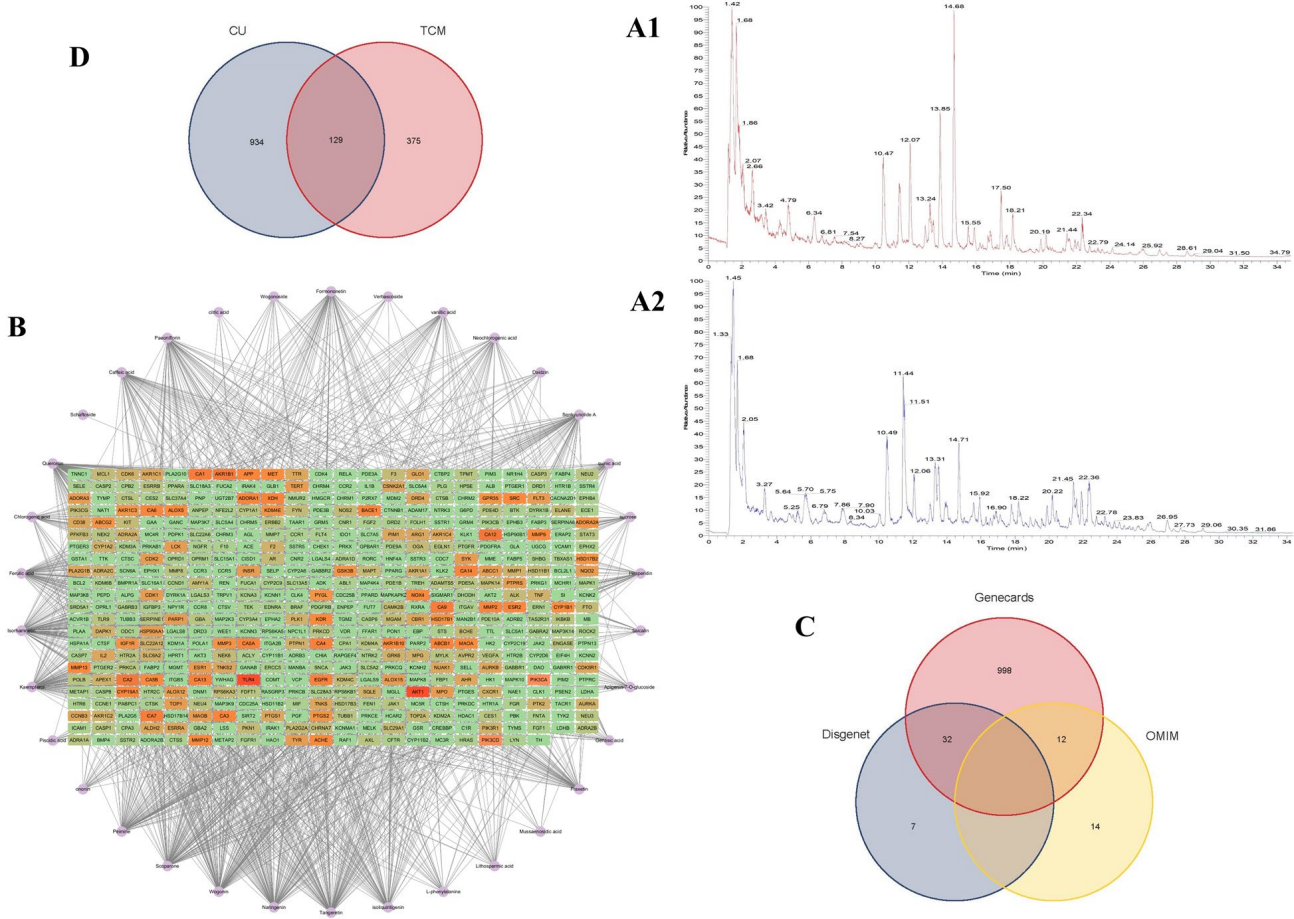
**A1** Base Peak Chromatogram (BPC) in ESI-mode, **A2** BPC in ESI + mode. **B** DGYZ-components-active ingredients-potential targets-pathways network. **C** Venn diagram of potential CU targets. **D** Venn diagram of potential targets of DGYZ and CU

Analysis of DGYZ’s active ingredients using HPLC-Q-TOF–MS identified 38 compounds, including 20 in positive ion mode and 18 in negative ion mode. These compounds were categorized as follows: 11 organic acids (e.g., citric acid, chlorogenic acid, caffeic acid), 10 flavonoids (e.g., kaempferol, quercetin, luteolin), 9 glycosides (e.g., paeoniflorin, swertioside, formononetin), 1 amino acid (L-phenylalanine), 3 terpenoids (e.g., peimine, cholic acid), and 4 other compounds (e.g., betaine, sucrose, isoliquiritigenin). Citric acid, betaine, paeoniflorin, kaempferol, and quercetin were most abundant based on peak area, indicating they are present in relatively high concentrations and may significantly contribute to DGYZ's pharmacological effects. These findings lay a strong foundation for further investigation into the relationship between DGYZ’s chemical composition and its pharmacological properties. The qualitative and quantitative data of the compounds are summarized in Table 1, and the total ion current chromatograms for both positive and negative ion modes are presented in Fig. 1A1, B2.

**Table 1:** DGYZ main active ingredient list

number	t_R_ (min)	Chemical formula	Ion mode	Theoretical value	Measured value	Error (× 10^–6^)	Secondary ion fragment information	Peak Area	Compound name
1	18.11	C_6_H_8_O_7_	–	191.0197	191.0194	− 1.5	111.0080;87.0078;57.0336	7357303425	Citric acid
2	1.38	C_5_H_11_NO_2_	+	118.0863	118.0865	1.7	59.0736;58.0658	2079539479	Betaine
3	16.85	C_23_H_28_O_11_	+	431.1337	431.1331	− 1.4	269.0809;213.0909;118.0418	1485074610	Paeoniflorin
4	3.66	C_15_H_10_O_6_	–	153.0193	153.0189	− 2.6	109.0287	570810058	Kaempferol
5	26.04	C_15_H_10_O_7_	+	193.1223	193.1225	1	175.1721;165.1278;137.0598	426705900	Quercetin
6	7.99	C_9_H_8_O_4_	–	179.035	179.0346	− 2.2	135.0445;107.0494;79.0542	361683047	Caffeic acid
7	6.8	C_15_H_14_O_6_	+	291.0863	291.0864	0.3	273.0765;181.0497;163.0392;153.0543;139.0391;111.0448	320058413	Catechin
8	9.98	C_15_H_14_O_6_	+	291.0865	291.0864	− 0.34	273.0761;181.0493;163.03885;139.0390;111.0442	293778135	Epicatechin
9	7.776	C_16_H_18_O_9_	–	353.0878	353.0884	1.7	191.0557;179.0346;135.0448	247308651	Neochlorogenic acid
10	4.26	C_16_H_18_O_9_	–	353.0878	353.0885	1.9	191.0057;179.0345;173.0451;161.0240;135.0443	223803949	Chlorogenic acid
11	14.65	C_29_H_36_O_15_	–	623.1981	623.199	1.4	461.1664;161.0239	186618087	Verminoside
12	4.35	C_16_H_24_O_10_	–	375.1297	375.1305	2.1	213.0768;169.0866;151.0758	140798214	Mussaenosidic acid
13	3.27	C_11_H_12_O_7_	–	255.051	255.0514	1.5	193.0504;179.0345;165.0552	133598219	Piscidic acid
14	2.48	C_9_H_11_NO_2_	–	164.0717	164.0714	− 1.8	120.0447;91.0542	95761521	L-phenylalanine
15	16.72	C_15_H_12_O_4_	+	257.0808	257.0806	− 0.8	257.0808;155.0342;137.0235	62616871	Isoliquiritigenin
16	12.31	C_21_H_20_O_9_	+	417.118	417.1187	1.6	255.0651;137.0234	56067135	Daidzin
17	12.69	C_10_H_10_O_4_	–	193.0506	193.0505	− 0.5	149.0602	54407363	Ferulic acid
18	15.42	C_28_H_34_O_15_	+	611.197	611.1976	1	431.1332;413.1230;345.0971;303.0864	49972882	Hesperidin
19	1.61	C_12_H_22_O_11_	–	341.1089	341.1092	0.9	179.0556;119.0342;113.0236;101.0236	40094997	sucrose
20	19.11	C_22_H_20_O_11_	–	459.0933	459.0944	2.4	283.0615;268.0380	39288083	Wogonoside
21	15.78	C_11_H_10_O_4_	+	207.0652	207.0653	0.5	192.0418;179.0704;164.0468;163.0390;151.0756	33414892	Scoparone
22	28.08	C_15_H_10_O_5_	–	269.0455	269.046	1.9	240.0437;225.0557;197.0609;183.1082	29318759	Aloe emodin
23	1.26	C_7_H_12_O_6_	–	191.0561	191.0557	2.1	173.0455;127.0392;85.0285;71.0129;59.0128	26372727	Quinic acid
24	15.4	C_15_H_12_O_5_	+	273.0757	273.0757	0	153.0183;119.0494	25213541	Naringenin
25	22.23	C_16_H_12_O_4_	+	269.0808	269.0809	0.4	254.0570;253.0500;226.0627;197.0599;137.0236	24863718	Formononetin
26	10.33	C_10_H_8_O_5_	–	207.0299	207.0297	− 0.96	161.0450;131.0340	20032488	Fraxetin
27	1.43	C_22_H_22_O_9_	–	1077.5123	1077.5141	1.7	915.4615;753.4066	15014346	Ononin
28	15.04	C_27_H_45_NO_3_	+	432.3472	432.3478	1.4	414.3368;301.8556	14228799	Peimine
29	12.35	C_26_H_28_O_14_	+	565.1552	565.1563	1.9	475.1018	13641684	Shaftaside
30	4.03	C_8_H_8_O_4_	–	167.035	167.0346	− 2.3	63.4389	9124338	Vanillic acid
31	13.48	C_16_H_12_O_7_	+	317.0656	317.0655	− 0.3	273.0396;245.0446;153.0183	8947182	Isorhamnetin
32	17.97	C_21_H_18_O_11_	+	447.0922	447.0922	0	271.0601	6812457	Baicalin
33	16.23	C_27_H_22_O_12_	–	537.1038	537.1048	1.9	295.0617;185.0241;109.0287	5185146	Lithospermic acid
34	15.16	C_21_H_20_O_10_	+	433.1129	433.1133	0.9	271.0602;153.0182	5095023	Apigenin-7-O-glucoside
35	19.08	C_16_H_12_O_5_	+	285.0757	285.0757	0	270.0524;242.0582;153.0181	4419599	Wogonin
36	21.29	C_7_H_6_O_4_	+	285.0404	285.0409	1.8	241.0508;211.0407;63.0231	4267692	Gentianic acid
37	25.74	C_20_H_20_O_7_	+	373.1282	373.1281	− 0.3	358.1038;343.0815;328.0568;300.0655;271.0600;211.0231;183.0288	2579488	Tangeretin
38	12	C_12_H_16_O_2_	+	303.0499	303.0502	1	285.0391;257.0443;229.0498;165.0184;153.0183;137.0234	2442177	Senkyunolide A

### NP-based analysis

#### DGYZ active ingredient-target network diagram

A network was then constructed to analyze the relationships between TCM and its target components. Network topology was evaluated by calculating betweenness, closeness, and degree centralities. The network diagram, consisting of 538 nodes and 1599 edges, highlights the multi-component and multi-target therapeutic interactions of DGYZ (Fig. 1B).

#### Prediction of CU targets

For disease target analysis, the Genecards, Disgenet, and OMIM databases were queried using the keyword “Chronic Urticaria.” Selection criteria included: Genecards targets with a relevance score ≥ 1; no screening for Disgenet due to limited targets; and only approved targets from OMIM. A total of 1,042 targets were identified from Genecards, 39 from Disgenet, and 26 from OMIM (Fig. 1C). Integration of these targets yielded 1,063 unique targets (Table 2).

**Table 2:** Compounds in the ingredient-target netwok of DGYZ (top 10)

Compound name	Molecular function	Degree	Betweenness	Closeness
Resveratrol	C_14_H_12_O_3_	366	400264.94	0.5029586
Quercetin	C_15_H_10_O_7_	145	97876.51	0.39098436
Dioscin	C_45_H_72_O_16_	112	92132.39	0.37117904
Emodin	C_15_H_10_O_5_	88	62526.574	0.37117904
Wogonin	C_16_H_12_O_5_	83	42169.52	0.37053183
Paeoniflorin	C_23_H_28_O_11_	65	2124.2541	0.3141713
kaempferol	C_15_H_10_O_6_	60	19085.078	0.3620102
7-O-methylisomucronulatol	C_17_H_18_O_5_	42	6508.1055	0.35475793
7-Methoxy-2-methyl isoflavone	C_16_H_12_O_4_	40	4198.828	0.35357738
Salidroside	C_14_H_20_O_7_	40	17756.287	0.34109148

#### The PPI network of DGYZ and CU common targets

**Fig. 2:**
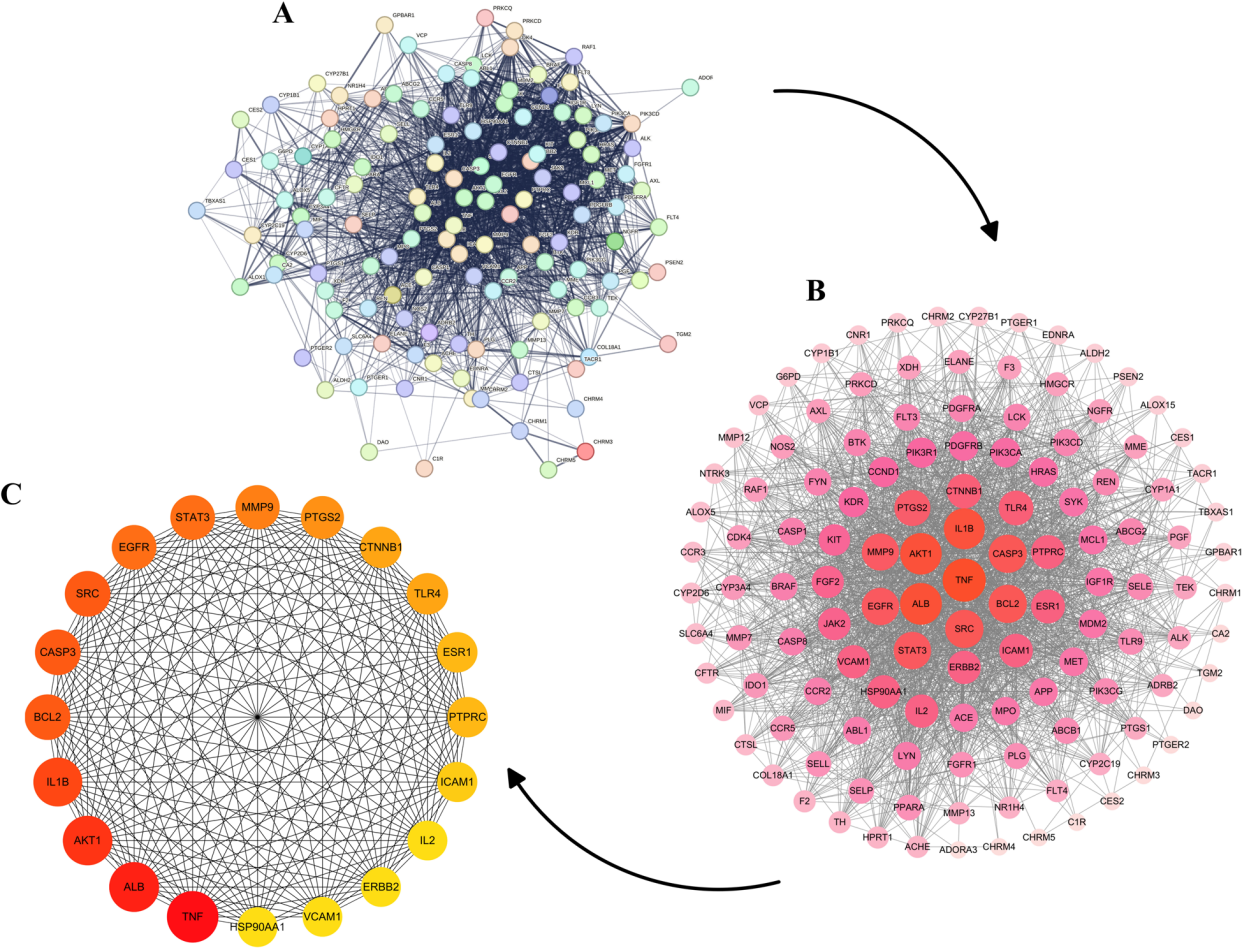
DGYZ and CU common target protein–protein interaction network. **A** PPI network of targets generated using STRING, Nodes represent proteins. Edges represent PPIs. **B** Visual analysis of the target using Cytoscape software, size and staining changes based on degree values. The darker the color is, the more significant the gene. **C**TOP20 targets selected by Degree value using cytohubba plugin. The darker the color is, the more significant the target

The intersection of active ingredients and disease targets was visualized using a Venn diagram (Fig. 1D). The 129 overlapping targets underwent PPI analysis using the String database, with a confidence threshold of 0.9 (Fig. 2A). The PPI network was visualized with Cytoscape, where node size and color represent degree values, with darker colors and larger circles indicating higher values (Fig. 2B, C). The top 20 targets, based on degree values, were identified using the cytohubba plug-in (Table 3).

**Table 3:** Top 20 in network string_interactions_short.tsv ranked by Degree method

Rank	Name	Scoreegree
1	TNF	40
2	AKT1	34
3	STAT3	32
3	IL6	32
5	SRC	31
6	IFNG	29
6	TP53	29
8	IL1B	28
9	EGFR	26
10	PIK3 CA	25
10	PIK3R1	25
12	IL10	24
13	PIK3 CD	22
14	TLR4	21
15	HSP90 AA1	20
15	CTNNB1	20
15	CXCL8	20
15	IL1 A	20
19	MAPK3	19
19	PTPN11	19

#### GO analysis and KEGG pathway enrichment analysis

**Fig. 3:**
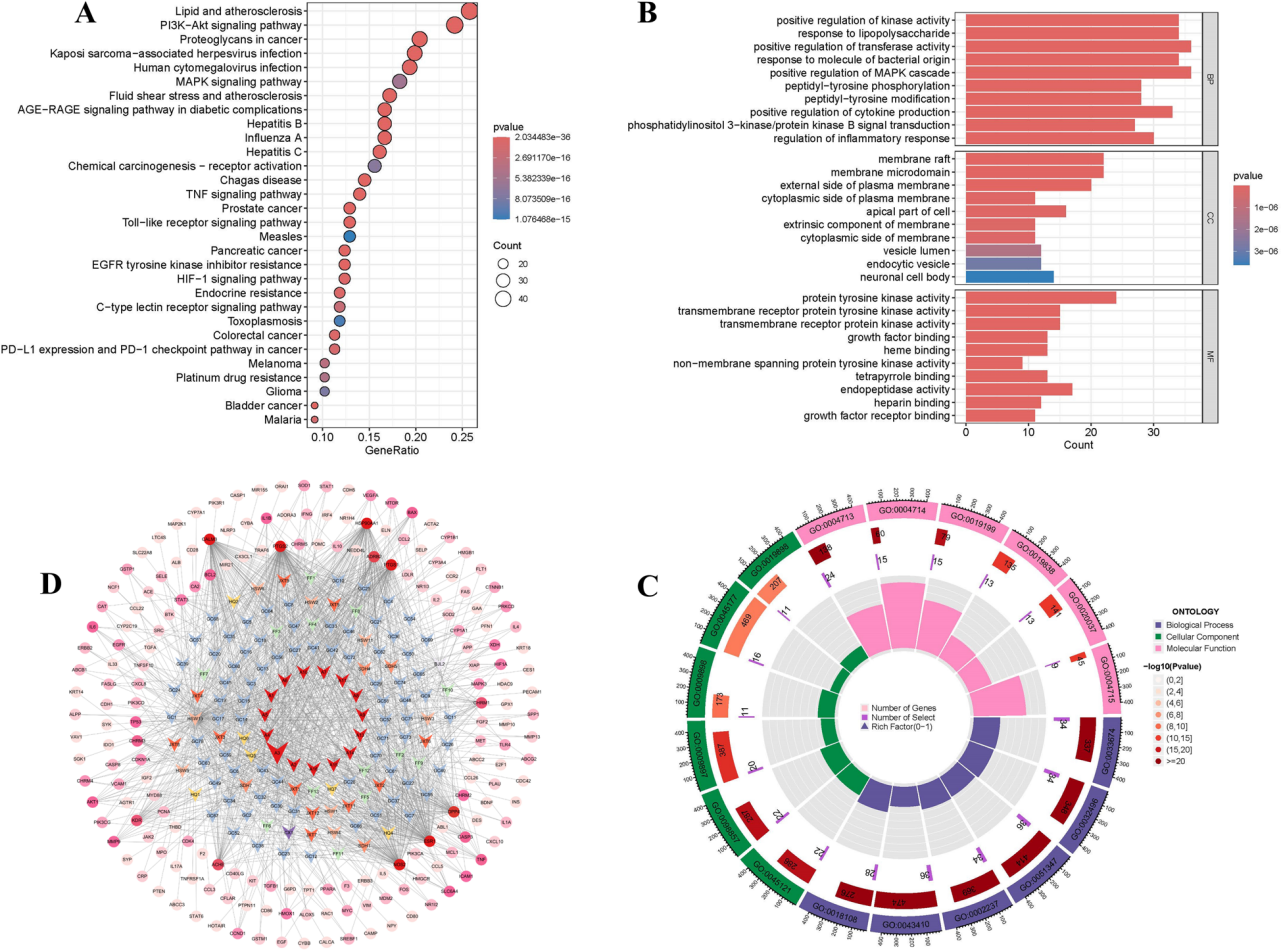
GO and KEGG pathway enrichment analysis results. **A** KEGG enrichment analysis. **B**, **C** BP, CC and MF enrichment analysis; X-axis represents the ratio of enriched target genes/background genes. Node color is displayed in a gradient from red to green in descending order of the P-value. The size of the nodes is arranged in ascending order of the number of genes. **D** The composition and target network of CU in PI3 K-AKT pathway treated by DGYZ decoction

Enrichment analysis was performed using R packages, including"DOSE,""clusterProfiler,""org.Hs.eg.db,"and"ggplot2,"focusing on KEGG and GO enrichment of the 129 intersecting targets. Key signaling pathways are summarized in Table 4. In KEGG pathway analysis, 161 signaling pathways were identified with P-values less than 0.05. The top five pathways were: Lipid and Atherosclerosis, PI3 K-Akt signaling pathway, Proteoglycans in Cancer, Kaposi Sarcoma-Associated Herpesvirus Infection, and Human Cytomegalovirus Infection. The bubble chart depicting the top 20 KEGG pathways is shown in Fig. 3A. The analysis revealed 102 CCs, 195 MFs, and 2,266 BPs with statistically significant differences (P < 0.05). The top five CCs with the lowest P-values were plasma membrane exterior, membrane raft, membrane microdomain, vesicle lumen, and cytoplasmic vesicle lumen, highlighting strong associations with cellular membranes. The top five MFs included cytokine receptor binding, cytokine activity, kinase regulator activity, DNA-binding transcription factor binding, and G protein-coupled receptor binding. For BPs, the top five processes were positive regulation of cytokine production, response to lipopolysaccharides, bacterial molecule response, positive regulation of the MAPK cascade, and positive regulation of transferase activity. Bar charts of the top 10 terms are shown in Fig. 3B and C.

**Table 4:** KEGG pathways of main pathway network of DGYZ Decoction (top 5)

KEGG pathway name	P value	Degree	p.adjust	qvalue
Lipid and atherosclerosis	2.03E-36	48	5.21E-34	1.76E-34
PI3 K-Akt signaling pathway	7.15E-23	45	3.05E-21	1.03E-21
Proteoglycans in cancer	1.01E-25	38	8.63E-24	2.91E-24
Kaposi sarcoma-associated herpesvirus infection	2.38E-25	37	1.52E-23	5.13E-24
Human cytomegalovirus infection	6.25E-22	36	2.00E-20	6.74E-21

#### Principal mechanism network of DGYZ in addressing urticaria

Cytoscape software (version 3.10.1) was used to visualize the enrichment of targets in the PI3 K-Akt signaling pathway. The network consisted of 160 nodes and 323 edges (Fig. 3D). In this diagram, the green rectangle represents the PI3 K-Akt signaling pathway, the pink circle represents the targets enriched in the pathway, and the orange diamond represents the TCM components related to these targets. The major nodes where the PI3 K-Akt pathway intersects with DGYZ components are detailed in Table 5.

**Table 5:** The PI3 K-AKT pathway axis and DGYZ component intersect the main node information

Node name	Degree	Betweenness	Closeness
HSP90 AA1	85.0	16461.854	0.60456276
PI3 K-Akt signaling pathway	45.0	8111.464	0.5824176
HSW13	33.0	880.6746	0.33194155
CHRM1	26.0	3557.8425	0.41732284
KDR	25.0	2813.8328	0.4151436
A3	15.0	929.9571	0.42857143
HSW3	15.0	1225.9832	0.43801653
A4	14.0	1086.8853	0.44289693
JXT1	14.0	813.64716	0.42627347
BCL2	13.0	549.3037	0.3906634

#### MD

**Fig. 4:**
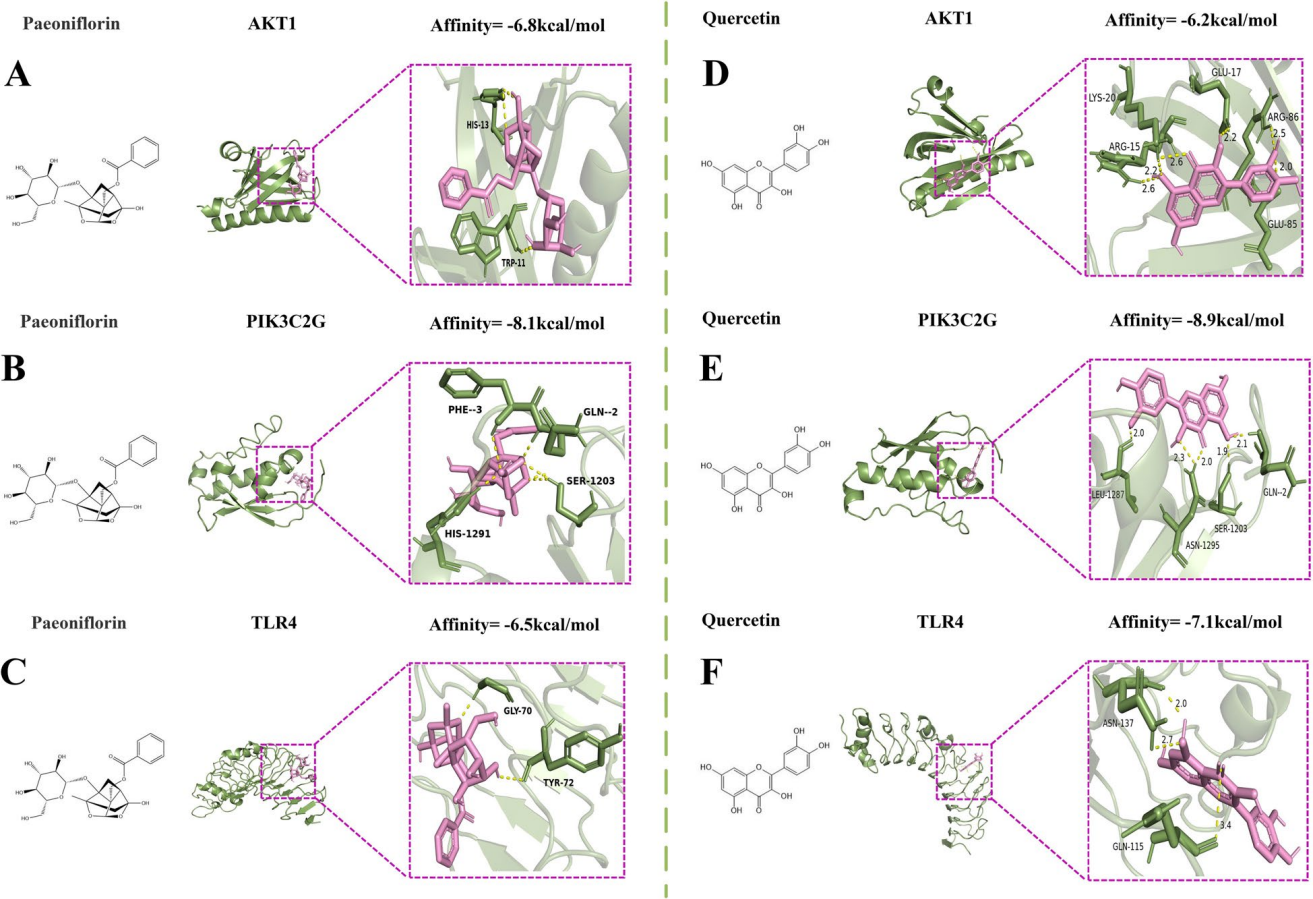
Molecular models of the binding of Paeoniflorin with (**A**) AKT1, (**B**) PI3 K3 C2G, (**C)**TLR4,and Quercetin with (**D**) AKT1, (**E**) PI3 K3 C2G, (**F**) TLR4 shown as 3D diagrams

To explore the interactions between Quercetin, Paeoniflorin, and the PI3 K-Akt signaling pathway proteins (PIK3 C2G, AKT1, and TLR4), DGYZ was used to predict the positions and grid box dimensions of protein binding sites (Table 6). Molecular docking was performed using AutoDock Vina software. The calculated binding affinities for the optimal binding poses are shown in Table 7. The results demonstrated good binding activity for all six groups, primarily through hydrogen bonding and hydrophobic interactions. Among these, Quercetin exhibited the strongest binding energy (− 8.9 kcal/mol) with PIK3 C2G, followed by Paeoniflorin with PIK3 C2G (− 8.1 kcal/mol). These findings are illustrated in Fig. 4.

**Table 6:** Target gene protein pocket coordinates and Grid box sizes

Target gene	PDB ID	Protein pocket coordinates	Grid box size
AKT1	1H10	X = 21.707, Y = 14.463, Z = 9.91	X = 40.5, Y = 39.0, Z = 45.75
PIK3 C2G	2 WWE	X = 33.373, Y = 20.486, Z = 9.076	X = 42.0, Y = 42.0, Z = 39.75
TLR4	2Z62	X = 13.988, Y = 0.115, Z = 8.276	X = 47.25, Y = 47.25, Z = 47.25

**Table 7:** The affinity value of the host ligand to the acceptor group

Target gene	Protein Affinity kcal/mol Compound	Docking energy
AKT1	Paeoniflorin	− 6.8
AKT1	Quercetin	− 6.2
PI3 K3 C	Paeoniflorin	− 8.1
PI3 K3 C	Quercetin	− 8.9
TLR4	Paeoniflorin	− 6.5
TLR4	Quercetin	− 7.1

### Animal experiment results

#### Protective effect of DGYZ on urticaria in mice

**Fig. 5:**
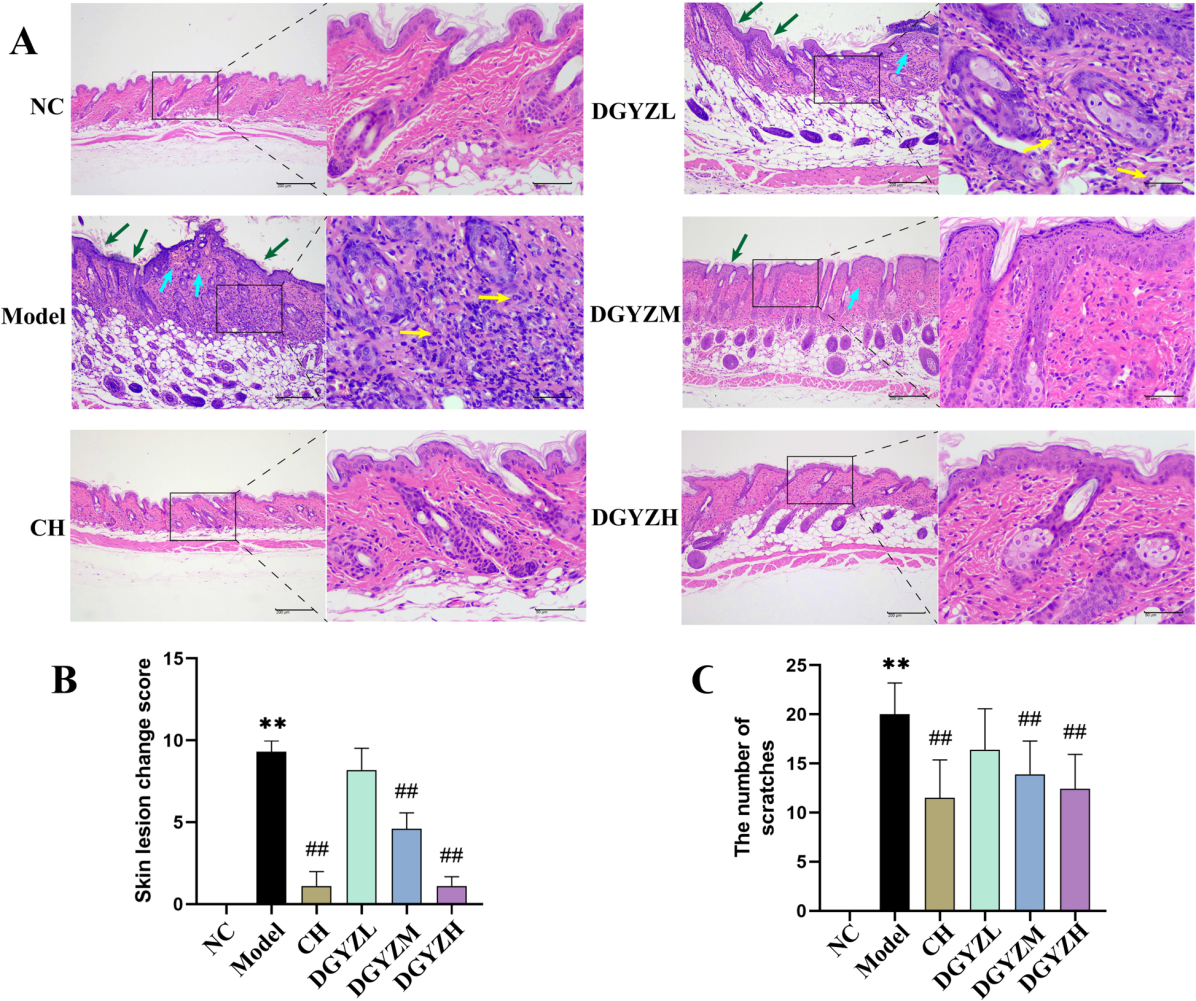
**A** DGYZ improved histopathology of urticaria. The back skin of mice in normal group, model group and DGYZ group was stained with hematoxylin and eosin (magnification, × 100, × 400). The green arrow points to the skin, the blue arrow points to dermal edema, and the yellow arrow points to inflammatory cells. **B **Comparison of skin lesion change score of CU mice. **C** Comparison of the number of scratches of CU mice.^*^P < 0.05, ^**^P < 0.01,Compared with normal group. ^#^*P* < 0.05, ^##^*P* < 0.01, compared with the model group

Oral administration of DGYZ significantly alleviated anti-DNP IgE-induced skin injury in mice. Hematoxylin and eosin (H&E) staining revealed that, compared to the NC group, the model group exhibited excessive epidermal keratosis, thickening of the granular layer, and dermal swelling and deformation. Additionally, capillary dilation and inflammatory cell infiltration were observed in the skin tissue of the model group. In contrast, DGYZ treatment improved skin damage in a dose-dependent manner, with results comparable to those in the CH (positive drug) group (Fig. 5A, B). In the scratching experiment, allergen-sensitized mice showed a significant scratching response. Compared to the model group, DGYZ treatment substantially reduced the number of scratches in a dose-dependent manner (Fig. 5C), confirming the significant protective effect of DGYZ on urticaria in mice.

#### DGYZ inhibits mast cell infiltration and degranulation.

**Fig. 6:**
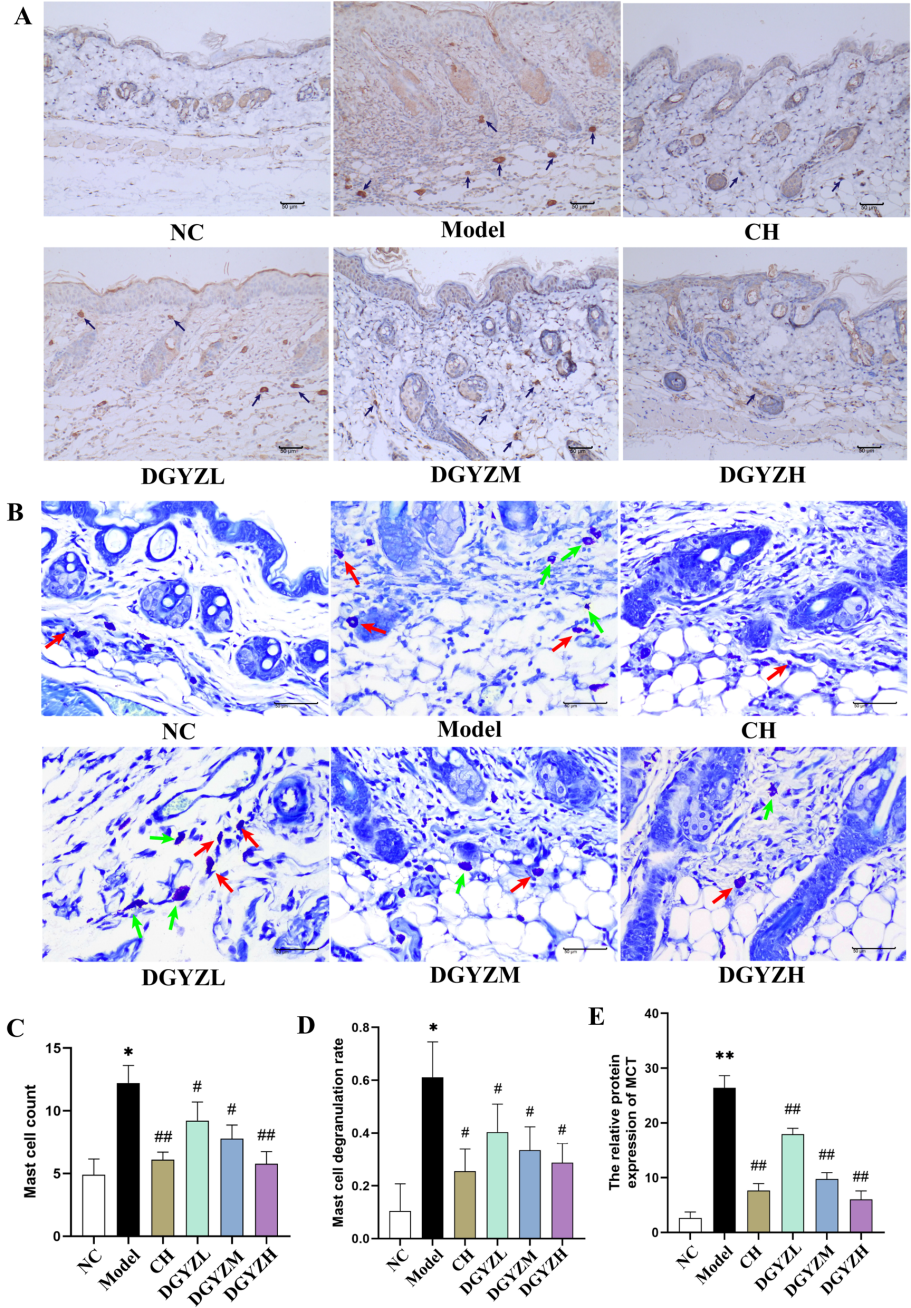
DGYZ inhibits mast cell infiltration and degranulation. **A** Representative MCT immunohistochemical images of mouse back skin (magnification, × 200). **B** Representative images of back skin mast cells of toluidine blue stained mice (magnification, × 400). **C** Comparison of mast cell count. **D** Comparison of mast cell degranulation rate. **E** Comparison of the raterelative expression level of MCT. ^*^P < 0.05, ^**^P < 0.01, Compared with normal group. ^#^*P* < 0.05, ^##^*P* < 0.01, compared with the model group

Mast cell activation and degranulation are central to Type I hypersensitivity reactions, a key process in urticaria. TB staining was performed to assess whether DGYZ inhibited mast cell activation. The number of mast cells and the extent of degranulation were significantly higher in the model group compared to the blank group (Fig. 6A, P < 0.01), confirming the occurrence of a Type I allergic response (Fig. 6B). However, following treatment with low, medium, and high doses of DGYZ, both the number of mast cells and the degranulation rate were significantly reduced in a dose-dependent manner (Fig. 6C, D, P < 0.05). Mast Cell Tryptase (MCT), a major component of mast cell secretory granules and a product of multiple genes, was also assessed by immunohistochemistry. MCT expression was markedly increased in the model group (P < 0.01), while treatment with high-dose DGYZ and CH significantly reduced MCT expression (P < 0.01, Fig. 6E). These findings further confirm that DGYZ suppresses mast cell activation and degranulation in a dose-dependent manner.

#### Impact of DGYZ on inflammatory factors, PI3 K/AKT and TLR4 signaling in pruritus mice

**Fig. 7:**
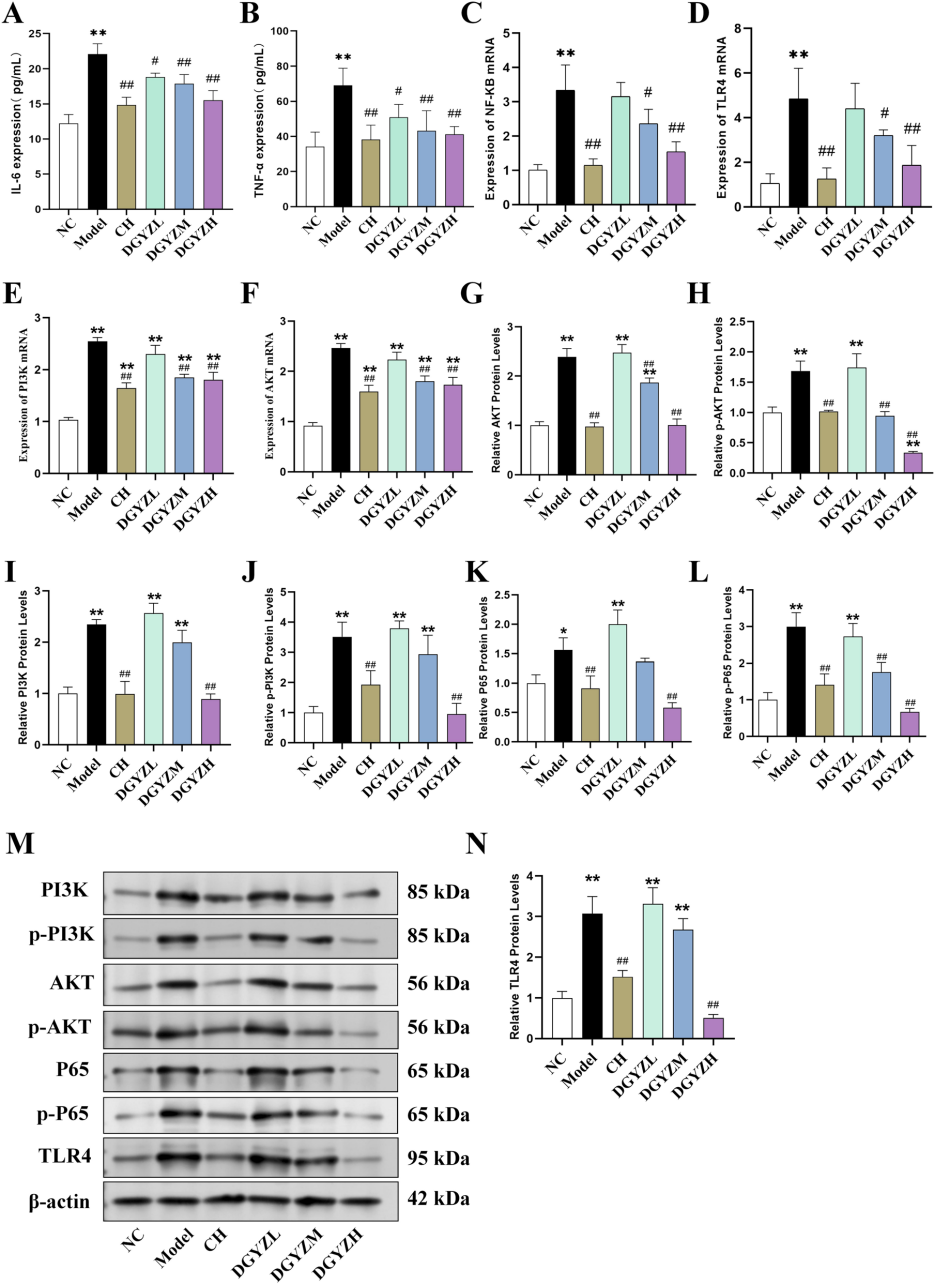
DGYZ decreased serum levels of (**A**) IL-6 and (**B**) TNF-α in mice with urticaria lesion. DGYZ treatment significantly decreased the expression of NF-κB mRNA, TLR4 mRNA, PI3 K mRNA and AKT mRNA (**C**–**F**). Western blot analysis showed the expression of P65, p-P65, PI3 K, p-PI3 K, AKT, p-AKT and TLR4 in dorsal skin of mice decreased after DGYZ intervention (**G**–**N**).^*^P < 0.05, ^**^P < 0.01, Compared with normal group. ^#^*P* < 0.05, ^##^*P* < 0.01, compared with the model group

Following successful sensitization, serum levels of mast cell degranulation-related factors IL-6 and TNF-α were significantly elevated in model mice, but oral administration of DGYZ effectively reversed these elevations (P < 0.01, Fig. 7A, B). Further investigation into inflammation-related signaling pathways revealed that DGYZ significantly inhibited the expression of components in the TLR4 signaling pathway, including TLR4 mRNA, NF-κB mRNA, PI3 K mRNA, and AKT mRNA (Fig. 7C–F). Protein expression levels of PI3 K, p-PI3 K, AKT, p-AKT, P65, p-P65, and TLR4 were notably higher in the model group compared to the control group, but treatment with DGYZ significantly reduced these levels (Fig. 7G–N), indicating the suppression of the PI3 K/AKT and TLR4 signaling pathways.

#### Effect of quercetin and paeoniflorin on mast cell degranulation and PI3 K/AKT and TLR4 signaling

**Fig. 8:**
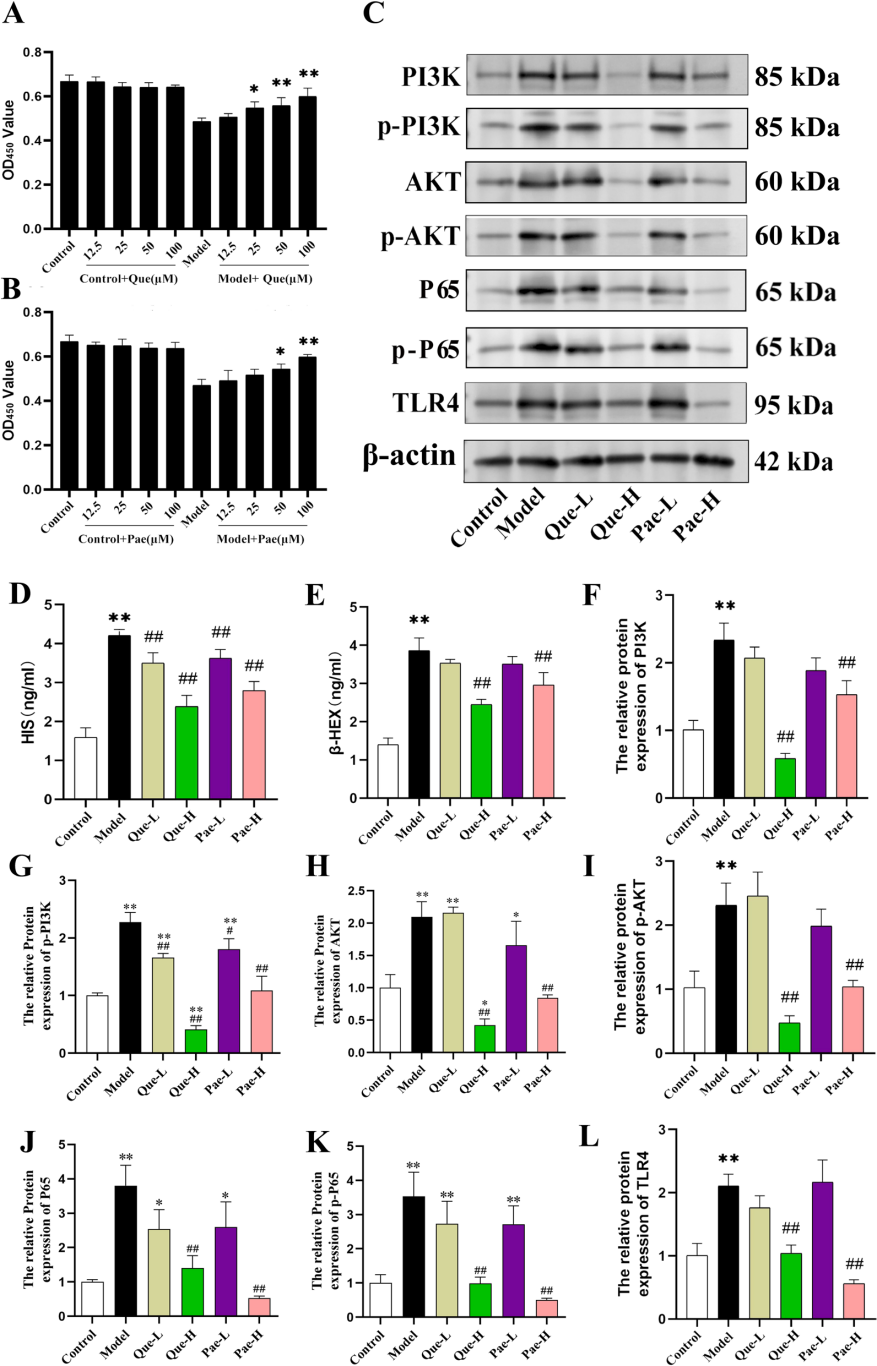
Maximum non-toxic dose screening of Paeoniflorin (**A**). Maximum non-toxic dose screening of Quercetin (**B**). The levels of β-HEX and HIS in model group cells were significantly higher than those in control group cells (**D**, **E**), and Quercetin and Paeoniflorin could significantly reduce the levels of β-HEX and HIS in model cells.Quercetin and Paeoniflorin inhibited the protein expression of AKT, p-AKT, PI3 K, p-PI3 K, P65, p-P65 and TLR4 in model cells(**C**, **F**–**L**). ^*^P < 0.05, ^**^P < 0.01, Compared with normal group. ^#^*P* < 0.05, ^##^*P* < 0.01, compared with the model group

The human mast cell line LAD2 was utilized to assess the effects of Quercetin and Paeoniflorin, primary active constituents of DGYZ. The maximum non-toxic doses of both compounds were determined, with no cytotoxicity observed at concentrations up to 100 μM. Based on these findings and previous research, 25 μM and 50 μM were selected as the low and high concentrations, respectively, for subsequent experiments (Fig. 8A, B). Subsequent analysis demonstrated a significant increase in β-HEX and HIS concentrations in model cells relative to controls, whereas treatment with Quercetin and Paeoniflorin substantially reduced these levels (Fig. 8D, E). Furthermore, both compounds significantly inhibited the expression of TLR4, AKT, p-AKT, PI3 K, p-PI3 K, P65, and p-P65 proteins in model cells (Fig. 8F–L).

## Discussions

TCM offers distinct advantages in the management of urticaria, especially in cases categorized under the"blood deficiency"pattern. In accordance with TCM principles, DGYZ is frequently prescribed for symptoms such as dry skin, pale complexion and lips, dizziness, pale tongue, and a weak pulse [33]. TCM theory posits that"Qi and blood"are the fundamental forces supporting human life, interdependent and mutually transformative. Qi regulates physiological functions and enhances immune defense, while blood serves as the material foundation for energy expenditure. Adequate Qi and blood are crucial for the proper functioning of all zang-fu organs, with their deficiency impairing immune function. Poor dietary and lifestyle habits can lead to the accumulation of"wet toxins,"which disrupt Qi and blood circulation, weaken immunity, and provoke hypersensitivity reactions such as wind-induced itching [13, 34, 35]. NP provides a systems biology approach to analyze the multi-target and multi-pathway mechanisms of TCM, clarifying the complexity of its components and their interactions with biological systems [36, 37].

Urticaria is characterized by wind masses and angioedema, often accompanied by severe itching. Clinically, patients may present with erythematous wheals of varying sizes and shapes, sometimes accompanied by systemic allergic symptoms [38]. While urticaria generally has a favorable prognosis, severe cases involving the respiratory or digestive tract may lead to serious complications such as dyspnea, vomiting, abdominal pain, or diarrhea [39].

Component-target network analysis revealed that GanCao exhibited the highest number of targets, followed by HeShouWu, JiXueTeng, and FangFeng. This analysis identified ten key compounds in DGYZ with the highest therapeutic potential. Notably, Resveratrol (RESV) has been reviewed for its antiallergic properties, including inhibition of mast cell (MC) degranulation, suppression of arachidonic acid derivative production, reduction of cytokine and chemokine expression, and blockade of pro-inflammatory signaling pathways [40]. Quercetin and Paeoniflorin significantly inhibit mast cell-dependent passive cutaneous anaphylaxis (PCA) in IgE-sensitized mice by suppressing IgE-mediated signaling pathways, including Syk, protein kinase C (PKC), phospholipase Cγ, p38, and c-Jun N-terminal kinase phosphorylation [41]. Quercetin also inhibits β-hexosaminidase and histamine release, calcium influx, and inflammatory factor expression in RBL-2H3 cells [42]. Dioscin reduces inflammatory cell infiltration and cytokine levels in bronchoalveolar lavage fluid (BALF) and lung tissue by lowering serum OVA-specific IgE/IgG1 levels and suppressing TGF-β1/Smad2/3 and protein kinase B (AKT) signaling pathways [43]. Similarly, Emodin inhibits cell proliferation and Akt activation in a dose-dependent manner, reducing airway smooth muscle cell (ASMC) proliferation by blocking the PI3 K/Akt pathway [44]. Wogonin downregulates the Th2 immune response induced by ovalbumin (OVA), particularly IgE and IL-5 expression, thereby exerting anti-allergic effects [45]. Paeoniflorin, a natural flavonoid primarily derived from the rhizome of Paeonia lactiflora, has been shown to reduce OVA/IgE-induced foot swelling in mice in a dose-dependent manner by inhibiting MC activation and lowering serum histamine, TNF-α, and IL-8 levels [46]. Salidroside has also been reported to alleviate inflammatory cell infiltration in OVA-induced asthma mouse models, suppress IL-4, IL-5, and IL-13 expression, and reduce phosphorylation of Akt and GSK3β [47].

A PPI network of shared targets for DGYZ and CU was constructed, with major gene functions analyzed using GO and KEGG enrichment. AKT1 emerged as a key target, and the PI3 K/Akt signaling pathway was identified as the most significant. This pathway plays a critical role in MC activation, a central mechanism in urticaria pathogenesis, characterized by MC activation and degranulation [48, 49]. Consistent with previous studies, AKT phosphorylation was significantly elevated in the urticaria model group, whereas treatment with DGYZ and CH effectively suppressed this process.

Urticaria is considered a mast cell-dominant disorder, wherein activated mast cells release histamine and other mediators, including leukotrienes, platelet-activating factors, and prostaglandins. These mediators induce vasodilation, plasma extravasation, and the activation of sensory nerves associated with pruritus [50]. Histamine is central to CU pathophysiology, while other inflammatory mediators, such as interleukin-6 (IL-6) and tumor necrosis factor-alpha (TNF-α), have been implicated in the severity of CU [51, 52]. IL-6, a key regulator of inflammation and immune responses [53], is significantly elevated in the serum of patients with CU compared to healthy individuals [54]. Moreover, treatment with omalizumab has been shown to decrease IL-6 levels in patients with CU [55]. MCT, which constitutes approximately 50% of the proteins secreted by mast cells during degranulation, is frequently used as a biomarker for mast cell activation [56]. Immunohistochemical analysis revealed a marked increase in mast cell activation and elevated MCT levels in the model group compared to the control group. Treatment with DGYZ or CH effectively reduced mast cell activation, with DGYZ showing a dose-dependent therapeutic effect.

The TLR4 and PI3 K/AKT pathways play crucial roles in the pathogenesis and treatment mechanisms of urticarial [57, 58]. TLRs, germline-encoded pattern recognition receptors (PRRs), play a central role in identifying pathogen-associated molecular patterns (PAMPs) and damage-associated molecular patterns (DAMPs) [59]. Among them, TLR-4 is a key receptor implicated in allergic diseases and autoimmune responses [60]. Microarray analyses have identified IL-6, TNF-α, NF-κB, and TLR-4 as pivotal genes in urticaria pathogenesis [61]. NF-κB, a critical regulator of inflammation and immune responses, promotes the production of pro-inflammatory cytokines that sustain CU symptoms [62]. This pathway also drives the recruitment of immune cells to inflammatory sites by activating the expression of immune-related genes [63]. Upon TLR4 ligand binding, MyD88 is activated, initiating downstream kinase production and nuclear translocation of NF-κB-related proteins. The activated NF-κB complex facilitates the transcription of immunoinflammatory response genes, leading to the release of pro-inflammatory cytokines such as TNF-α, IL-1β, and IL-6 [64]. The PI3 K/Akt molecular pathway plays a pivotal role in the pathogenesis of CU. Its mechanisms in the treatment of CU mainly involve the following aspects: it participates in and regulates the activation and degranulation of mast cells [71]. Through upstream and downstream regulatory mechanisms, it influences the autophagy cell cycle, metabolism, and promotes cellular oxidative stress [65]. Moreover, it enhances the release of allergen-specific IgE inflammatory mediators [49]. As a canonical regulatory pathway for eosinophil-related inflammatory factors, the downstream molecules of the PI3 K/Akt pathway are implicated in multiple oxidative stress-related pathways, such as those associated with IgE and TNF-α, thereby modulating the inflammatory response. The development of CU is closely associated with pathological alterations, including mast cell activation and imbalances in inflammatory cytokines, which are induced by abnormal signal expressions within the PI3 K/Akt pathway. Therefore, investigations into this pathway are of great significance for the clinical remission of CU [32, 66].

This study identified Quercetin and Paeoniflorin as the primary active components of DGYZ. Previous studies have shown that these compounds effectively inhibit IgE-mediated allergic reactions [67]. Specifically, Quercetin suppressed histamine overproduction in paclitaxel-activated RBL-2H3 cells in a dose-dependent manner in vitro [68]. In agreement with these findings, mast cell degranulation induced by DNP-BSA was observed in the human mast cell line LAD2, with treatment using 50 μM of Quercetin and Paeoniflorin significantly inhibiting this process. The underlying mechanism is likely related to the suppression of PI3 K/AKT phosphorylation and TLR4 signaling pathways. Quercetin enhances SHP-1 phosphorylation and inhibits the MyD88/IKK/NF-κB signaling pathway. It also inhibits F-actin through the PI3 K/AKT/Rac1/Cdc42 pathway, reduces MRGPRX2 internalization, and thus inhibits mast cell degranulation [69, 70]. Paeoniflorin has the ability to inhibit the PI3 K/Akt/mTOR signaling pathway, leading to a reduction in the expression levels of BAFF, BAFF-R, PI3 K, p-Akt and mTOR. This inhibitory effect suggests that paeoniflorin holds potential for treating or improving immune-related disorders typified by urticaria. The diverse active components in DGYZ do not operate in isolation; instead, they likely interact through a synergistic and potentiating mechanism. Quercetin predominantly alleviates allergic reactions by impeding the release of β-hexosaminidase, histamine, and inflammatory factors. Conversely, paeoniflorin is chiefly engaged in suppressing mast cell activation. In the in-vivo context, these components might modulate the immune response with great precision by targeting distinct elements within key signaling pathways like PI3 K—Akt and TLR4. This synergy confers upon DGYZ a more potent immunomodulatory effect compared to its individual components. Notwithstanding, the specific synergistic model and the underlying molecular mechanisms necessitate further in-depth exploration.

To further elucidate the mechanism of DGYZ, changes in the expression of Akt, P-AKT, NF-κB, p65, and TLR4 were analyzed in an animal model of urticaria. The PI3 K/Akt signaling pathway, which plays a crucial role in IgE-induced mast cell degranulation, was hypothesized to mediate the therapeutic effects of DGYZ by suppressing both the PI3 K/Akt and NF-κB pathways. While the findings of this study have advanced the understanding of the mechanisms underlying CU treatment, several limitations must be addressed. Although NP predicted interactions between Quercetin, Paeoniflorin, and key targets, the lack of experimental validation of binding patterns restricts the depth of mechanistic insight. Future research should employ techniques such as surface plasmon resonance to confirm binding affinities and elucidate the molecular interactions of these compounds with their targets. Furthermore, despite highlighting the importance of the PI3 K/Akt axis in DGYZ-mediated CU improvement, the absence of loss-of-function studies, such as PI3 K/Akt knockdown assays, prevents definitive conclusions regarding its indispensability. Incorporating these experiments into future investigations would validate the pathway’s role more conclusively. Additionally, the lack of transcriptomic analysis in CU models treated with DGYZ limits understanding of its broader effects on biological pathways, including PI3 K/Akt. Employing transcriptome sequencing technologies in future research could uncover molecular network alterations induced by DGYZ, offering a comprehensive overview of its pharmacological actions. Lastly, the failure to identify active drug components in the blood limits insights into DGYZ’s in vivo pharmacokinetics and bioactivity. Future studies should focus on isolating and characterizing these constituents to better understand their roles in CU treatment and enhance the theoretical foundation for therapeutic applications.

## Data Availability

The data used in this study can be provided in accordance with requests. If you need the relevant data for academic research or other legitimate purposes, please contact the corresponding author. We will respond to your request in a timely manner and provide the data following proper procedures.

## Abbreviations

CU: Chronic urticaria
DGYZ: Dang-Gui-Yin-Zi
UPLC-Q-TOF–MS: Ultra-performance liquid chromatography coupled with quadrupole time-of-flight tandem mass spectrometry
PPI: Protein–protein interaction
C-T-D: Compound-target-disease
GO: Gene ontology
KEGG: Kyoto encyclopedia of genes and genomes
MD: Molecular docking
DNP-IgE: Anti-2,4-dinitrophenyl-specific IgE
DNFB: 2,4-Dinitrofluorobenzene
TNF-α: Tumor necrosis factor-α
TLR4: Toll-like receptor 4
NF-κB: Nuclear factor-κB
PI3 K: Phosphoinositide 3-kinase
Akt: Protein kinase B
IL-6: Interleukin-6
OD: Optical density
IHC: Immunohistochemistry
WB: Western blot
RT-PCR: Real-time polymerase chain reaction
PCA: Passive cutaneous anaphylaxis
RESV: Resveratrol
MC: Mast cell
PKC: Protein kinase C
PLCγ: Phospholipase Cγ
p38: P38 Mitogen-activated protein kinase
JNK: C-Jun N-terminal kinase
OVA: Ovalbumin
MCT: Mast cell tryptase
CIU: Chronic inducible urticaria
CSU: Chronic idiopathic urticaria
NP: Network pharmacology
ESI: Electrospray ionization
BP: Biological process
CC: Cellular component
MF: Molecular function
H&E: Hematoxylin and eosin
TB: Toluidine blue
IgE: Immunoglobulin E
HIS: Histamine
β-HEX: β-Hexosaminidase
BSA: Bovine serum albumin
ASMC: Airway smooth muscle cell
TLR: Toll-like receptor
PRRs: Pattern recognition receptors
PAMPs: Pathogen-associated molecular patterns
DAMPs: Damage-associated molecular patterns
MyD88: Myeloid differentiation primary response 88
Syk: Spleen tyrosine kinase
